# Survival, movements, home range size and dispersal of hares after coursing and/or translocation

**DOI:** 10.1371/journal.pone.0286771

**Published:** 2023-06-02

**Authors:** Neil Reid

**Affiliations:** School of Biological Sciences, Queen’s University Belfast, Belfast, Northern Ireland, United Kingdom; University of Cordoba, SPAIN

## Abstract

Hare coursing is the pursuit of a hare by dogs for sport. In recent years in Ireland, between 2,900 to 3,700 hares have been caught from the wild (under Government license) and held in captivity for up to 8 weeks. Hares are given a head start and coursed in an enclosed arena by two muzzled greyhounds where the object is not to kill the hare, but judge the dogs on their ability to turn the hare which escapes under a partition through which the dogs cannot follow. Recent licence returns suggest over 99% of hares survive and are released back into the wild. This study aimed to assess survival and behaviour of coursed hares after their release sometimes into unfamiliar territory. Forty hares were tracked using GPS-radio collars for six months after release in a factorial experimental design to test the impact of coursing and translocation on survival, movements, home range size and dispersal. Coursed and uncoursed hares did not differ in observed mortality rates, movements, home range sizes or dispersal distances after release back into the wild though fewer coursed than uncoursed hares were relocated six months after release, due to a combination of collar strap failures and radio silence. Spatial behavior was similar between the cohorts once translocated hares, which moved further and had larger home range sizes during the first four days after release, had settled. Two hares released shortly before sunset were killed in road traffic collisions during their first night. Releasing hares during daylight, preferably as early as possible, may provide time for animals to settle before darkness. Suggestions are made for potential methodological improvements such as the use of cellular (mobile phone) or satellite communication technology mounted on stouter straps to reduce failures and improve relocation rates.

## Introduction

Hare coursing is the pursuit of a hare by long dogs. Coursing can be ‘open’, where the hare is chased *in situ* being flush from its form (its daylight lie-up site) or ‘closed’, where it is caught and transported to a coursing field. In Europe, coursing occurs in Spain and Portugal and notably Ireland. The Irish hare (*Lepus timidus hibernicus*) is a game or quarry species and can be hunted during an open season from the 26^th^ September to the 28^th^ February each year [1]. Unlike in other European countries, hares are not traditionally shot in Ireland but instead are hunted on foot with a pack of harrier hounds or, more popularly, coursed using greyhounds. The Irish Coursing Club (ICC) consists of up to 90 local coursing clubs distributed all over the country but concentrated in the south. In recent years (2020/21 to 2022/23), between *ca*. 2,900 to 3,700 hares have been captured from the wild each year under Government licence by long netting [2]. Nets 1.5m tall are hung from forked sticks to create a pouch at ground level. These are placed across likely exit routes from an area to be walked over by a line of beaters such that hares flushed from their daily forms run into the net [3]. Target areas include unimproved rough pasture with substantial cover of rushes, *Juncus* species, in which hares shelter during daylight hours. Hares are ear tagged for individual identification, boxed and transported to a local paddock. Captive hares are given about a 75m head start before being pursued by two muzzled greyhounds within an enclosed football field-sized arena where the object is not to kill the hare but turn it from a straight course. The dog responsible for turning the hare (identified as the one wearing either a white or red collar) wins the match. The average course lasts less than 40 seconds [4]. Regional heats and tournaments are held with the best dogs competing at a National meeting. Dogs are selectively bred with their pedigree having been documented for many generations. Hares escape through a barrier or partition at the end of the coursing field through which the dogs cannot follow before being returned to the paddock. Throughout up to 8 weeks in captivity, hares are supplementary fed with, for example, oats and grain, willow, apples etc. and receive veterinary treatment including being dosed with a broad spectrum anthelminthic such as Ivermectin, to rid them of parasitic ticks, lice and worms [4] and Baycox® (Toltrazuril) to prevent coccidiosis. The introduction of compulsory dog muzzling, and the sharing of best practice in captive hare husbandry between clubs, improved survival [4] with license returns suggesting over 99% of hares are typically released back into the wild [5]. Coursing licences state that “*the same numbers of hares must be released back into the wild as far as possible at the same locations from which such numbers were captured*” though this may result in hares being inadvertently translocated and released into unknown territory provided a similar number are released, as was caught, at each site. Habitually the same sites are used year-on-year for the capture and release of hares and they are often owned and managed by farmers who are members of a local coursing club. As such, habitat suitable for hares such as rough and semi-improved grassland may be left agriculturally unimproved to maintain hare numbers locally. Predators such as foxes may be controlled, for example, by shooting while other forms of hare hunting may be prohibited locally. Consequently, so-called hare preserves, may be associated with a higher hare population density than comparable areas in the wider countryside [6].

What happens to coursed hares after their release back into the wild remains largely unknown [7] though one previous study radio-tracked nine coursed hares demonstrating survival up to 11 weeks after release (the end of the study) but there were no control animals and no analysis of the effects of translocation [8]. This study aimed to assess survival and behaviour of coursed hares over six months after their release back into the wild. The specific objectives were to compare: i) relocation rates and ii) movements, home range sizes and dispersal distances between coursed and uncoursed, and, translocated and untranslocated hares to examine their impacts both in isolation, and together. These data may help inform Government in decision making when granting licences for hares to be taken from the wild.

## Methods

### Ethical statement

The proposed research protocol was peer-reviewed by two academics working in the field of animal behaviour who reported to the Faculty of Medicine, Health and Life Sciences Research Ethics Committee at Queen’s University Belfast who approved the proposal on 24^th^ March 2021 (Faculty REC Reference No. MHLS 20_124 amended 4^th^ February 2022). The Irish Coursing Club were not involved in experimental design including sample size determination, data collection, analysis, or interpretation. Queen’s University Belfast was not responsible for, or otherwise involved in, the coursing of hares with already coursed hares being collared just before their release back into the wild. The Irish Coursing Club were licenced by NPWS under Ministerial approval to capture live hares under Section 34 of the Wildlife Act 1976 to 2018 (as amended) and to attach a tag to a wild animal under Section 32 (see www.npws.ie/licencesandconsents/hare-coursing). To facilitate the current research, an additional condition was added to the licence stating that “*the ICC and its affiliated clubs shall co-operate with* […] *the study*” which was supported willingly and with full prior consent. Uncoursed hares were netted from the wild. The author was licenced to capture protected wild animals for scientific purposes under the Wildlife Act Sections 23 and 34 (Licence No. C55/2021) and to attach a tag to a wild animal under Section 32 (Licence No. 05/2021). NPWS Conservation Rangers, Kieran Brennan and Hugh McLindon, inspected hare handling, collaring and release procedures.

### Experimental design

A fully factorial 2x2 experimental design was used with two interacting treatments. Treatment 1 consisted of 20 coursed and 20 uncoursed hares, Treatment 2 consisted of 20 translocated and 20 untranslocated hares with both treatments interacting such that half of each overlapped with the other i.e. 10 translocated coursed, 10 untranslocated coursed, 10 translocated uncoursed and 10 untranslocated uncoursed hares, were used (the latter group being the wild control group). This approach was adopted to tease apart the potential impacts of coursing and translocation in isolation and together. Any ear tags were removed the day before release. In a full experimental design, some might expect that uncoursed hares should be subject to all treatments e.g. transport, ear tagging and captivity, with the exception of coursing but the intention here was not to disaggregate the effects of coursing from other associated activities but rather to compare coursed hares (subject to transport, ear tagging and captivity etc.) to wild animals. The total sample size (40 hares) was restricted by the logistical considerations of how many animals could be tracked simultaneously and funding. The sample size exceeded the number of individual hares GPS-radio tagged in other recent studies of wild hare behaviour (for example, 25 hares in [9]; 32 hares in [10] and 34 hares in [11]).

Twenty hares had been coursed by the Irish Coursing Club in the normal operation of the National Coursing meeting at Clonmel, Co. Tipperary in early February 2022. Coursed hares spent up to 8 weeks in captivity. Hares were collared the day after the National meeting with 10 released at their original site of capture and 10 released into unfamiliar territory at a site other than where they were captured. Translocated coursed hares were relocated between counties (many tens of kilometres from their capture site) having been brought from other coursing districts. A further twenty hares were captured from the wild using long nets. These hares were temporarily held in boxes (for a few hours) until the full cohort had been caught before each was collared with 10 released at their site of capture and 10 released into unfamiliar territory. Translocated uncoursed hares were relocated locally with a distance between capture and release of >3 to 10km (previous radiotracking of Irish hares reported a maximum recorded dispersal distance of *ca*. 2km; [12]). Translocated uncoursed hares were taken across a river *ca*. 5 metres wide with few crossing points. No translocated animals dispersed from their release site back to near their point of capture. A total of 34/40 hares (85%) were collared and released on the 8^th^ February 2022 (including both coursed cohorts) with the remaining six hares (15%—all uncoursed) caught from the wild and released four days later on 12^th^ February 2022.

### Animal tagging

**Fig 1:**
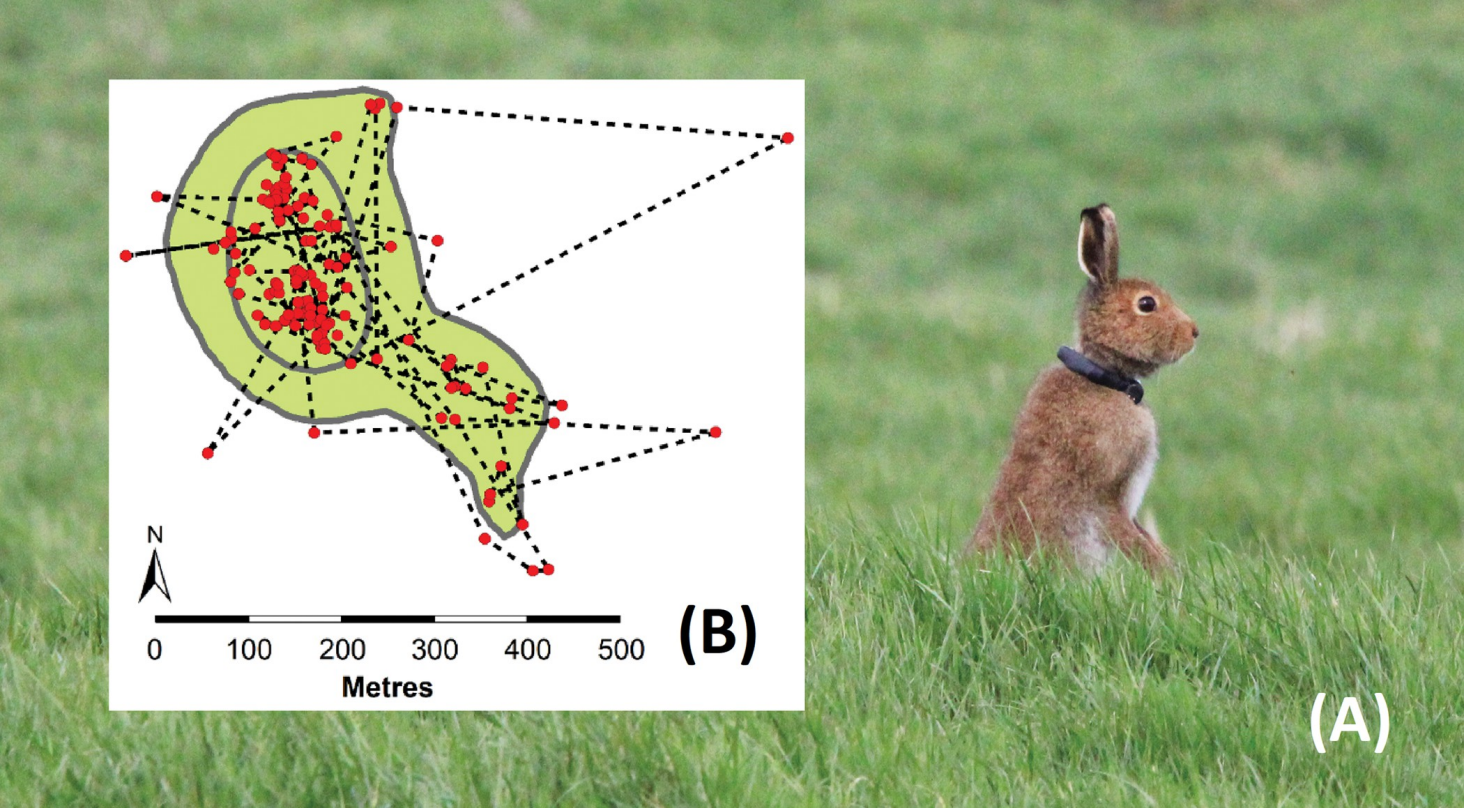
A tagged hare with an example of movements and home range. (A) An Irish hare (*Lepus timidus hibernicus*) wearing a LOTEK Ltd. Litetrack RF60 collar after release back into the wild. Photograph © Neil Reid. (B) An example of a hare’s GPS locations for one week (red dots), 95% kernel home range (outer polygon), 50% kernel core range (inner polygon), and interfix step length movements (dashed lines). In this example, the average step length was 68m/hr, the furthest single movement was 530m with the animal travelling on average 1.7km/24hrs and 11.6km/week.

Hares were tagged with Litetrack RF60 GPS collars (Fig 1A) supplied as a single made-to-order batch from LOTEK Ltd. (www.lotek.com/products/litetrack-60). All animals were weighed to the nearest 100g. GPS collars were approx. 63g which was on average ~2% (range = 1.4–2.3%) of the body weight of tagged hares which was on average 3.4kg (range = 2.8–4.5kg, *n* = 40). Collars were programmed to collect a SWIFT GPS fix once every hour such that battery life and storage capacity would enable collection of a minimum of six months of spatial data. Each collar had a mortality sensor such that should it remain motionless for 24 hours it recorded the date and time motionlessness started whilst the rate of the radio signal beeps doubled alerting the researcher enabling recovery. Collar VHF (very-high frequency) radio beacons were programmed to be active 10am-5pm GMT daily to enable animals to be detected, triangulated and their data downloaded using an UHF (ultra-high frequency) radio channel activated from about 50m away. Data downloads occurred during daylight hours when animals were lying up in their forms undisturbed.

### Collar testing

The operation and programming of each collar was initially tested for 24 hours prior to deployment. Collars were hung from wooden pegs 30 centimeters above ground level replicating their approximate height and orientation when attached to a sitting hare. Pegs were placed in a 6x7 grid with 3 metres spacing between collars in an improved grassland field. The coordinates for their actual positions were measured out on the ground relative to fixed features e.g. a wall and hedge line such that their positions could be accurately mapped using Google Earth Pro aerial imagery georeferenced in ArcMap 10.8 (ESRI, California, USA). All VHF signals were confirmed as detectable using a Sika radio receiver and flexible yagi antennae (www.lotek.com/products/biotracker-vhf-receiver). All mortality beacons were confirmed to activate after 24 hours of no motion. GPS fixes (1 per hour) were downloaded from each collar using the UHF radio channel to confirm remote data download was operational.

The distance between each GPS fix and the known coordinates of each collar was calculated using the Near function in the Spatial toolbox for ArcMap. After exclusion of three fixes with errors 13-32km from the test grid (fixes where only 2–3 satellites were found), collars had an average error of 7 metres with an average maximum error of 39 metres per collar and maximum single GPS error of 264m. Thus, even when collars were motionless (replicating a hare lying up during daylight) there was apparent movement usually in the region of tens of metres per hour attributable solely to GPS locational error. As all collars were equally subject to this source of error its effects are unlikely to have biased the results between experimental cohorts.

All collars were cleared of test data and deactivated until just before deployment. During actual deployment on an animal, each collar was activated and its radio beacon confirmed as signalling before attachment.

### Tracking

After release, animals were relocated by triangulation of their daily VHF beacons after one week with spatial data downloaded. Animals were subsequently relocated with data downloaded once per month up to the 18^th^ August 2022 (191 days or 6.3 months after release).

Experience in the field suggested that collar VHF beacons were undetectable much beyond *ca*. 350 metres. During each visit, the extent over which hares ranged as observed from data downloaded on any previous visit was walked whilst scanning through all collar radio frequencies to find animals if they were not immediately detectable from their last known position. All minor roads, and many farmers lanes and tracks, were driven at 10–15 kph out to *ca*. 5km from each release site with a roof-mounted omnidirectional antennae with continuous scanning of all missing collar radio frequencies. Sites were further searched from the air on 3^rd^ June 2022 by privately chartered helicopter. A yagi antenna was fixed to one of the helicopter skids pointing directly behind the aircraft. A spare collar was placed on the airfield at a known location and the aircraft flown at the lowest allowable altitude of 500ft (152m) directly away from the collar until the signal was lost at *ca*. 800–1,000m suggesting detection distances may have been up to three times further from the air than ground-based surveys. Sites were flown circling outward. Any potential signals detected from the air were investigated further from the ground (both by vehicle and on foot) immediately after landing.

### Outcomes

The fate of each tagged hare was classified at the end of the study as: i) alive (relocated at 6 months), ii) dead (carcass or collar retrieved with evidence of death e.g. animal-vehicle collision, predation), iii) unknown, which was further broken down into: iv) never relocated after release, v) relocated with subsequent loss of radio signal, vi) collar retrieved having been slipped overhead still bolted closed and vii) collar retrieved after strap failure. Variation in the proportion of hares classified by outcome was tested across experimental cohorts using 2x2 or 2x4 χ2 Contingency tests with the former comparing two cohorts (each with 20 hares) and the latter four cohorts (each with 10 hares). The probability of relocating a hare throughout the six months was compared between cohorts using Kaplan-Meier survival analysis and Log Rank Mantel-Cox tests. Variation in sex ratios between cohorts was examined using 2x2 χ2 Contingency tests.

### Movements

GPS fixes were cleaned to remove single points falling well beyond each study site (more than tens of kilometres) indicative of GPS error associated with too few satellites being used for triangulation.

Hare movement was measured as step lengths i.e. the distance between successive hourly GPS locations. Collars failed to log GPS coordinates on 12% of hourly fixes due to too few satellites being detected. Movements were, thus, standardised per hour. That is, the distance between points two hours apart was divided by two, and so on, to express movement as a rate in metres per hour (m/hr).

Hare activity patterns (average hourly movements across the 24-hour period) was described by a boxplot of step lengths with variation analysed using a Generalized Linear Mixed Model (GLMM) fitting Hour of the day as a Fixed Factor and HareID as a Random Factor to account for repeated measures of each animal throughout the study. Sunrise and sunset times varied from 4-8am and 5-9pm respectively between February during mid-winter (initial release) and June during mid-summer (four months later). Thus, not only was it necessary to adjust for daylight savings (BST to GMT) but also to standardise any activity profile relative to date-specific local sunrise and sunset creating a daylight and darkness offset. Hourly movements were described as a boxplot of step lengths offset relative to sunrise and sunset (both adjusted to a value of 0) with daylight activity given as the number of hours into daylight as a positive integer (up to local midday) and activity in darkness given as the number of hours into darkness as a negative integer (up to local midnight). Based on the observed activity pattern, the 24-hour period was subsequently split into an Active period and Inactive period.

It was not simply a matter of adding all consecutive step lengths together to get a measure of the total distance travelled by each individual due to the problem of GPS failure. To account for missing data, the average mean value for each hare’s rate of movement, in metres per hour, for each day’s Active and Inactive periods was calculated and multiplied by the number of hours in that day’s Active and Inactive periods. The sum of the Active and Inactive estimates provided a total daily estimate. This multiplicative method essentially filled in the average movements for missing values specific to each day, for each hare, and allowed the total distance covered by each hare, every 24 hours, to be estimated. To avoid the creation of any artefacts by imputing missing data, the estimated total distance travelled each day was linearly regressed with the summed total distance travelled each day for hares where all 24-hourly fixes were obtained i.e. those days when there were no GPS failures as sufficient satellites had been found for each fix. These were near perfectly correlated (r^2^ = 0.994; S1 Fig) with only a 0.5 metre average difference between the estimated and true value out of 1.7 km/24hrs (i.e. estimated values were within 0.03% of the true values). Thus, this method was used to estimate total distances covered by hares when not all hourly movement values were present.

Total estimated daily movements were analysed every day over the first week after release to examine immediate effects of experimental treatments. Total estimated distance travelled every 24 hours was fitted as the dependent variable in a GLMM with Day fitted as a continuous covariate and sex, coursing, translocation and their interaction as fixed factors and HareID fitted as a Random Factor. This analysis was repeated and restricted to the first 4 days, and last 3 days, of the first week. Two further models were fitted, one restricted to the Active period and another to the Inactive period with identical model structure.

Total estimated daily movements were also analysed every week over the 27 weeks (6.3 months) during which spatial data were collected to examine longer-term effects. GLMMs of total estimated distance travelled every 24 hours were fitted again but this time with Week as a continuous covariate and sex, coursing, translocation and their interaction as fixed factors and HareID fitted as a Random Factor. Again, this analysis was repeated and restricted, this time to Weeks 2–15 i.e. excluding Week 1 and Weeks 16–27. Two further models were fitted, one for the Active and another for the Inactive period with identical model structure.

### Home range size

Spatial analyses were conducted using the program Ranges 9 v4.01 (Anatrack Ltd, England www.anatrack.com). A hare’s home range i.e. the extent of the area used by the animal expressed in hectares, was defined as the 95% kernel polygon enclosing GPS fixes while their core range was defined as the 50% kernel (Fig 1B). These were drawn using subsets of the data to produce daily home ranges for the first week, and, weekly home ranges over the duration of the study. Home range size was fitted as the dependent variable in GLMMs with either Day or Week fitted as a continuous covariate as appropriate and sex, coursing, translocation and their interaction fitted as fixed factors with HareID as a Random Factor.

### Dispersal

Ranges 9 software estimated the distance between the release site of each animal and each of its subsequent GPS locations. This distance was fitted as the dependent variable in a GLMM with Week fitted as a continuous covariate and sex, coursing, translocation and their interaction as fixed factors with HareID as a Random Factor.

## Results

### Outcomes

**Fig 2:**
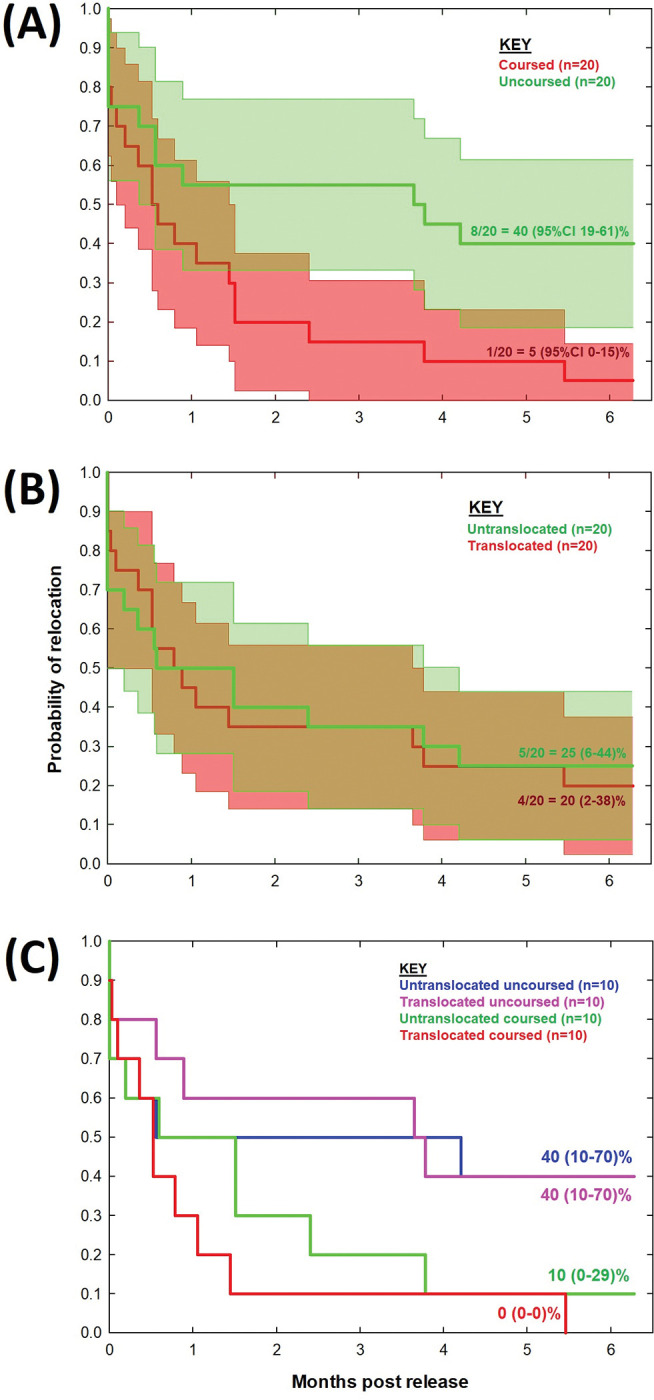
Hare relocation rates over six months after release. Kaplan-Meier plots showing declining probability of relocating a hare (solid lines) ± 95% Confidence Intervals (shading) comparing (A) coursed and uncoursed and (B) translocated and untranslocated hares. These two cohorts (*n* = 20 hares in each) were further broken down into (C) four cohorts (*n* = 10 in each): untranslocated uncoursed, translocated uncoursed, untranslocated coursed and translocated coursed. For simplicity, 95% Confidence Intervals have not been shown in (C) but are reported as text next to each line.

Fewer coursed than uncoursed hares were relocated at six months after release (5% compared to 40% respectively); not because observed mortality rates differed (which were 10% and 5% respectively) but because more coursed than uncoursed hares were undetected at the end of the study (85% compared to 55% respectively) with their fate unknown. Decline in hare relocation rates was more rapid for coursed than uncoursed hares (Kaplan-Meier χ^2^_df = 1_ = 4.032, *p* = 0.045, Log Rank Mantel-Cox χ^2^_df = 1_ = 5.811, *p* = 0.016) with the 95% Confidence Intervals of relocation at six months after release between 0–15% for coursed hares and 19–61% for uncoursed hares (Fig 2A). There was no difference in survival, mortality or unknown outcomes between translocated and untranslocated cohorts (Table 1). Relocation rates over the six months were similar between translocated and untranslocated hares (Kaplan-Meier χ^2^_df = 1_ = 0.004, *p* = 0.949, Log Rank Mantel-Cox χ^2^_df = 1_ = 0.058, *p* = 0.810) with the 95% Confidence Intervals of relocation six months between 2–38% for translocated hares and 6–44% for untranslocated hares (Fig 2B). The translocated coursed cohort was the only group to have no animals remaining near the release site five and a half months later (Fig 2C). Unknown outcomes were partly attributable to 50% of translocated coursed hares removing their collar by scratching the strap until it wore through (S2 Fig) within, on average, 16 days (range 0–32 days); a behaviour not observed in any other cohort. Collar strap failure was not associated with coursing as none of the untranslocated coursed hares wore through their collar straps and it was not associated with translocation as none of the translocated uncoursed hares did so either. The decline in the rate of relocation was more rapid for untranslocated coursed hares than either uncoursed cohort (whether translocated or not; Fig 2C) with more having unknown outcomes as six months (80%) than any cohort (Table 1).

**Table 1:** Outcomes for 40 collared hares after six months. Values are individual counts of hares. Statistical differences between cohorts were tested using a 2x2 or 2x4 χ2 Contingency tests as appropriate for two or four cohorts respectively. Asterisks highlight outcomes that differed significantly (*p*<0.05) between cohorts. ∞ indicates a complete difference in outcome i.e. that outcome occurred in one cohort only.

Outcome	Treatments																	
	Coursing					Translocation					Coursing*Translocation							
	Coursed	Uncoursed	χ^2^	Df	*p*	Translocated	Untranslocated	χ^2^	df	*p*	Translocated coursed	Untranslocated coursed	Translocated uncoursed	Untranslocated uncoursed	TOTAL	χ^2^	df	*p*
Alive—relocated at 6 months	1	8	7.03	1	0.008*	4	5	0.14	1	0.705	0	1	4	4	9	5.16	3	0.160
Dead—carcass or evidence of death	2	1	0.36	1	0.548	2	1	0.36	1	0.548	2	0	0	1	3	0.83	3	0.843
Unknown	17	11	4.29	1	0.038*	14	14	0.00	1	1.000	8	9	6	5	28	4.76	3	0.190
**TOTAL**	**20**	**20**				**20**	**20**				**10**	**10**	**10**	**10**	**40**			
**Breakdown of Unknown**																		
Never located after release	3	4	0.17	1	0.677	2	5	1.56	1	0.212	0	3	2	2	7	1.46	3	0.691
Relocated with subsequent loss of radio signal	7	4	1.13	1	0.288	5	6	0.13	1	0.723	2	5	3	1	11	4.39	3	0.222
**SUB-TOTAL–**Undetected at six months	**10**	**8**	0.40	1	0.525	**7**	**11**	1.62	1	0.204	**2**	**8**	**5**	**3**	**18**	8.48	3	0.037*
Collar slipped overhead—still bolted closed	2	3	0.23	1	0.633	2	3	0.23	1	0.633	1	1	1	2	5	0.69	3	0.877
Collar strap failed—worn through	5	0	∞	1	∞	5	0	∞	1	∞	5	0	0	0	5	∞	3	∞
**SUB-TOTAL—**Collars retrieved	**7**	**3**	0.21	1	0.144	**7**	**3**	0.21	1	0.144	**6**	**1**	**1**	**2**	**10**	9.07	3	0.028*

Five out of forty hares (12.5%) removed their collars by slipping them over their heads (Table 1) still bolted closed indicating too loose a fit with animals removing collars on average 38 days (range 0–128 days) after release. The collar of one untranslocated uncoursed hare was reported by a farmer as being found among straw used as cattle bedding after opening a round bail. The collar was closed and intact with no apparent evidence of predation or death (no blood/flesh/hair). The morality sensor indicated that movement stopped 4.2 months after release within an improved grassland field within the animal’s core home range. The collar lay motionless for 4 days before a single sudden 730m movement to the farmyard where its signal was eventually tracked some weeks later. How it found its way into a straw bail could not be determined but there was no evidence that the animal had died by either predation or agriculture (it was thus recorded as a collar removal rather than a death).

Two hares (one translocated coursed and one untranslocated uncoursed) were killed in animal-vehicle collisions occurring on a National and Regional road within 460m and 425m from their respective release sites. Mortality sensors indicated that one was killed 2.5hrs and the other 8.7hrs after release. At one location, a roadkill fox (*Vulpes vulpes*), otter (*Lutra lutra*) and stoat (*Mustela erminea*) were also found within 300m of the recovered collar along a straight stretch of National road with notably fast traffic. One roadkill hare was scavenged by a fox four days after death and carried 620m away to be buried in the bank of a stream. The collar of a translocated coursed hare was recovered recording mortality 5.5 months after release with the collar still bolted closed with evidence of either predation or scavenging with fox tooth marks evidencing chewing on the external rubber housing and associated blood and flesh.

Due to collar retrievals and loss of signals, coursed hares were tracked for half the time of uncoursed hares with on average 40 compared to 97 days of spatial data respectively (S1 Table). After the last spatial data were collected 6.3 months after release, the Irish Coursing Club recaptured three of the studied hares in the course of their normal netting operations during October and November 2022 (all three were uncoursed with two translocated and one untranslocated). These animals were alive 8.0 to 9.5 months after release. Two of these animals had been tracked for the full six-month study, but one had been last located in April. Thus, records were updated to recode the outcome for this individual from unknown to alive at six months.

Outcome for hares at six months was unrelated to their original body weight (Kruskal-Wallis_df = 4_ = 3.665, p = 0.453; S3 Fig). Sex ratios whilst male biased (1.7:1 male:female) did not differ between coursed and uncoursed (χ^2^_df = 1_ = 0.107, *p* = 0.744), and, translocated and untranslocated (χ^2^_df = 1_ = 0.107, *p* = 0.744) cohorts (S1 Table).

### Activity

**Fig 3:**
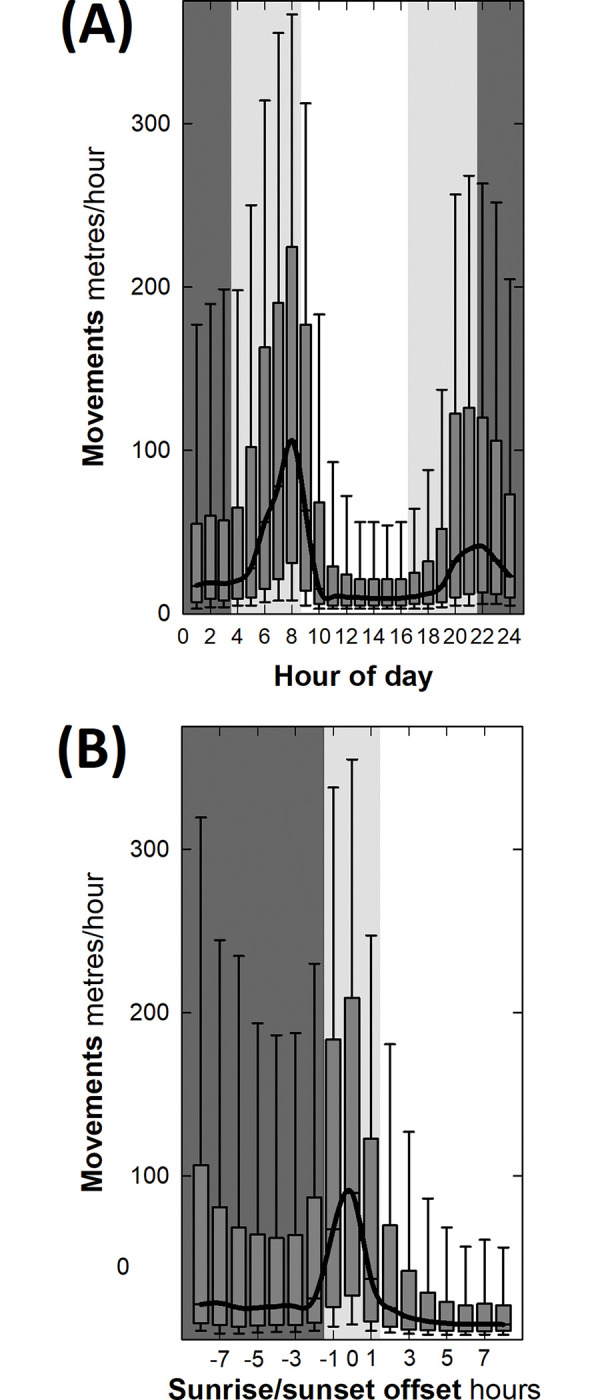
Hare activity throughout 24 hours. Boxplot (median, interquartile range and 95% Confidence Intervals) of hare movements in metres per hour i.e. step lengths between successive GPS fixes throughout (A) the 24-hour period showing nocturnal (dark grey), crepuscular (light grey) and diurnal activity (no shading). The earliest and latest sunrise and sunset times varied from 4-8am and 5-9pm between mid-winter (initial release in February) and mid-summer (four months after release during June) respectively. (B) shows the same but with daily activity folded over date-specific local sunrise and sunset (both represented by 0) with hourly offsets during night shown as negative integers and day as positive integers adjusted for daylight savings (BST to GMT). This removes the drifting of sunrise and sunset times and standardised activity relative to the date-specific light-dark cycle.

Hourly movements of hares varied throughout the 24-hour period (*F*_df = 23, 54843_ = 227.95, *p*<0.001; Fig 3A). Hares spent most of the day motionless in their forms with any apparent movement likely attributable to GPS error. Accounting for the drift in daily sunrise and sunset times, hares were most active between the hour before and after sunrise and sunset (Fig 3B) being 2.5 times as active at sunrise as sunset (Fig 3A). Thus, the active period of the day was defined as one hour before sunset until one hour after sunrise and the inactive period as one hour after sunrise until one hour before sunset.

### Daily movements during the first week

**Fig 4:**
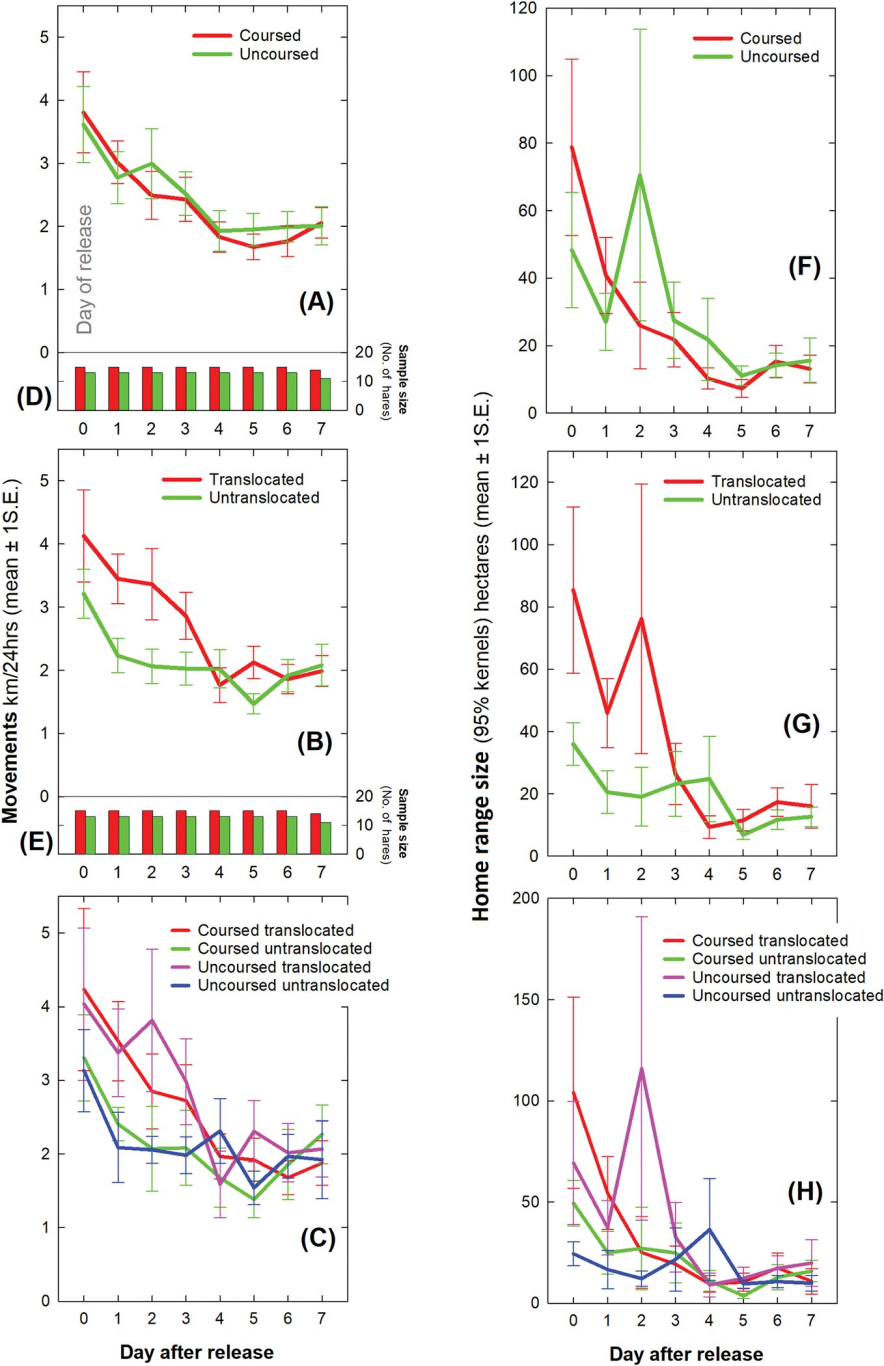
Average daily hare movements throughout the first week after release. Kilometres per 24 hours ± 1 standard error comparing (A) coursed and uncoursed, (B) translocated and untranslocated and (C) all four cohorts of hares. Samples sizes in individual hares for (D) coursed and uncoursed and (E) translocated and untranslocated hares. Home range size (the extent of 95% kernel polygon) in hectares comparing (F) coursed and uncoursed, (G) translocated and untranslocated and (H) all four cohorts of hares.

The distance travelled by hares decreased over the first week (Days 0–7: *F*_df = 7,209_ = 10.128, *p*<0.001) from an average of 3.6 (95%CI: 3.1–4.2) km during the day of release (Day 0) dropping over the next 4 days before stabilizing thereafter (the average of Days 4–7 was 1.9 (1.6–2.3) km/24hrs). There was no difference between the sexes (*F*_df = 1,209_ = 0.043, *p* = 0.835) or coursed and uncoursed hares (*F*_df = 1,209_ = 0.046, *p* = 0.830; Fig 4A). Translocated hares moved further than untranslocated hares during the first 4 days only (Days 0–3 average for translocated hares was 3.4 (2.7–4.1) km/24hrs which was 1.5 times further than untranslocated hares at 2.3 (1.5–3.1) km/24hrs (Days 0–3: *F*_df = 1,106_ = 4.121, *p* = 0.045; Fig 4B). However, translocated hares settled by Day 4 with no difference between the movements of either group thereafter (Days 4–7: *F*_df = 1,103_ = 0.015, *p* = 0.904; Fig 4B). There was no interaction effect between coursing and translocation i.e. no difference between any of the four cohorts (Coursing*Translocation: *F*_df = 1,209_ = 0.040, *p* = 0.842). Translocated coursed and translocated uncoursed cohorts had greater movements during the first 4 days i.e. translocation had the same effect on coursed and uncoursed hares (Fig 4C). Samples sizes of the number of hares contributing spatial data each day were comparable between cohorts throughout the first week (Fig 4D & 4E).

Daily active period movements (dusk, night and dawn) declined through the first week (Day: *F*_df = 1,215_ = 54.426, *p*<0.001) from 3.4 (2.9–3.9) km on the day of release (Day 0) to an average of 1.7 (1.3–2.0) km/active period on Days 4–7 reflecting the majority of the variation in the 24-hourly pattern described above. There were no effects of sex, coursing, translocation or their interaction on apparent movements during what was otherwise the inactive period.

### Daily home range size during the first week

In common with daily movements, daily home range size (95% kernel/24hrs) decreased in area throughout the first week (Day: *F*_df = 1,215_ = 16.877, *p*<0.001) from an average of 60 (38–84) hectares during Day 0 to an average of 14 (10–17) ha/24hrs during Days 4–7 (Fig 4F). There were no effects of sex (*F*_df = 1,215_ = 0.364, *p* = 0.547) or coursing (*F*_df = 1,215_ = 0.090, *p* = 0.764). Translocated hares had an average home range size of 68 (33–103) ha during the first three days (Days 0–2), which was substantially greater than untranslocated hares with an average of 18 (1–59) ha (Days 0–2: *F*_df = 1,78_ = 3.543, *p* = 0.064; Fig 4G). Thereafter, home range size was similar between translocated and untranslocated hares (Days 3–7: *F*_df = 1,131_ = 0.007, *p* = 0.934). Home range size did not vary between the four cohorts during the first week (Coursing*Translocation: *F*_df = 1,215_ = 0.280, *p* = 0.597; Fig 4H). Its notable that untranslocated uncoursed hares (the wild control group) had the lowest variation in daily home range size during the first week (the blue line in Fig 4H). Patterns in daily core range size (50% kernel/24hrs) were the same as 95% kernels and are not reported for simplicity.

A single individual translocated uncoursed hare (a male) made a notably unusual and very large exploratory movement over the course of the night during Day 2. During this excursion, it crossed a river twice (outbound and inbound journeys) in locations where there were no bridges and navigated throughout unfamiliar territory circumnavigating the entire study site before returning to near its release site. This accounted for the peak in home range size during Day 2 in the translocated and uncoursed cohorts (green line, Day 2, Fig 4F; red line, Day 2, Fig 4G and pink line, Day 2, Fig 4H). Similarly, a single untranslocated uncoursed hare (also a male) moved away from the release site during the night of Day 4 before returning accounting for the smaller peak in this cohort (blue line, Day 4, Fig 4H). Removal of both individuals from the sample resulted in relatively smooth declines in movements and home range size throughout the first week but their movements were real and not GPS artefacts, and as such, there was no *a priori* basis for their removal and they were thus retained in analysis.

### Movements over six months

**Fig 5:**
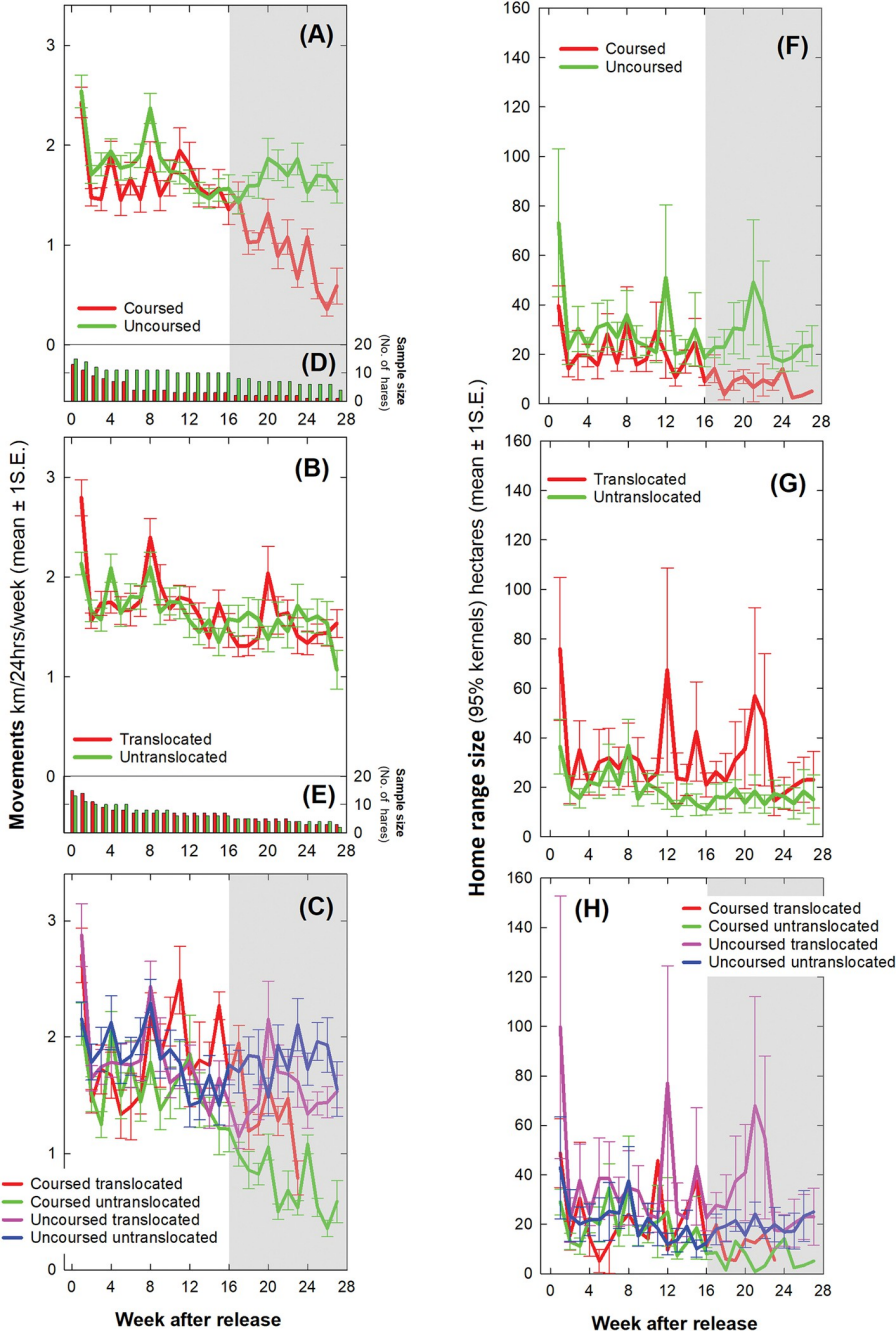
Average daily hare movements per week over six months after release. Kilometres per 24 hours per week ± 1 standard error comparing (A) coursed and uncoursed, (B) translocated and untranslocated and (C) all four cohorts of hares. Sample sizes for (D) coursed and uncoursed and (E) translocated and untranslocated hares. Home range size (the extent of 95% kernel polygon in hectares) comparing (F) coursed and uncoursed, (G) translocated and untranslocated and (H) all four cohorts of hares. The shaded area from Weeks 16–27 indicates that caution should be taken in interpreting results due to a small sample size of coursed hares contributing spatial data.

The average daily distance covered by hares each week decreased throughout the six-month study (Week 1–27: *F*_df = 1,2492_ = 154.986, *p*<0.001) with a notable drop between Week 1 (the week of release) and Week 2 with a marginal decline in activity thereafter (Fig 5A–5C). There was no difference between the sexes (*F*_df = 1,2492_ = 0.235, *p* = 0.628), coursed and uncoursed hares (*F*_df = 1,2492_ = 0.222, *p* = 0.637; Fig 5A), translocated and untranslocated hares (*F*_df = 1,2492_ = 1.117, *p* = 0.291; Fig 5B) or their interaction (*F*_df = 1,2492_ = 0.654, *p* = 0.419; Fig 5C) throughout the six months. The average daily distance covered by a hare was 1.7 (1.4–2.0) km/24hrs/week. Movements of coursed hares appeared to decline markedly after Week 16 (Fig 5A) but this reflected a decline in the number of individual hares contributing spatial data which had dropped to two individuals in the coursed group compared to 8 individuals remaining in the uncoursed group by Week 16 (Fig 5D). To be conservative, the data were re-analysed excluding Week 1 and Weeks 16–27 i.e. excluding initial movements including the disturbance of handling, tagging and release and the period of poorest sample size towards the end of the study. During Weeks 2–15, coursed hares moved 1.5 (1.1–1.9) km/24hrs/week which was about 200m less than uncoursed hares at 1.7 (1.4–2.0) km/24hrs/week but this difference was not statistically significant (*F*_df = 1,1576_ = 0.919, *p* = 0.338) and well within the variation observed between individuals.

The ratio of the duration of active-to-inactive periods each 24-hours shifted from the release of hares in early-to-mid February during winter with 17 hours of activity (dusk, night and dawn) and 7 hours of inactivity (daylight) per day up to the summer solstice during late June with 7 hours of activity and 17 hours of inactivity per day. After the solstice the ratio shifts back toward more hours of darkness. Average daily movements during active and inactive periods showed an effect of Week (Week 1–27: *F*_df = 1,2493_ = 546.942, *p*<0.001 and *F*_df = 1,2447_ = 162.137, *p*<0.001 respectively) with active movements shortening (from 2.2km/active period in Week 1 to *ca*. 522-680m/active period throughout most of the summer months) and inactive movements lengthening (from 281m/inactive period in Week 1 to 820m/inactive period in Week 20 when the solstice occurred) before drifting back in the other direction again. There were no effects of sex, coursing, translocation or their interaction on either active or inactive period movements as reflected by the 24-hourly analysis above.

### Weekly home range size over six months

Weekly home range size (95% kernel/wk) decreased in area throughout the six months study (Week: *F*_df = 1,351_ = 8.930, *p* = 0.003), initially from an average of 55 (41–69) ha during Week 1 to 15 (1–30) ha by Week 2 (Fig 5F–5H). Home range size was unaffected by sex (*F*_df = 1,351_ = 0.986, *p* = 0.321) or coursing (*F*_df = 1,351_ = 0.845, *p* = 0.359; Fig 5F). Translocated hares had an average weekly home range size of 32 (19–45) ha which was substantially larger than untranslocated hares with home range size of 15 (1–30) ha (*F*_df = 1,351_ = 2.801, *p* = 0.095); a weak but persistent effect throughout the six months (Fig 5G). There was no interaction between coursing and translocation (*F*_df = 1,351_ = 0.137, *p* = 0.711; Fig 5H).

In common with movements, average home range size declined markedly in the coursed group after Week 16 reflecting the drop in sample size of individual hares contributing spatial data (Fig 5D). Re-analysis excluding Week 1 and Weeks 16–27 suggested no change in home range size between Week 2 to Week 15 (Weeks 2–15: *F*_df = 1,220_ = 0.074, *p* = 0.785). Weekly home range size in Weeks 2–15 were unaffected by sex (*F*_df = 1,220_ = 1.644, *p* = 0.201), coursing (*F*_df = 1,220_ = 0.577, *p* = 0.448), translocation (*F*_df = 1,220_ = 1.713, *p* = 0.192) or their interaction (*F*_df = 1,220_ = 0.464, *p* = 0.497). There were no effects of sex, coursing, translocation or their interaction on weekly core range size (50% kernel/wk).

### Dispersal

**Fig 6:**
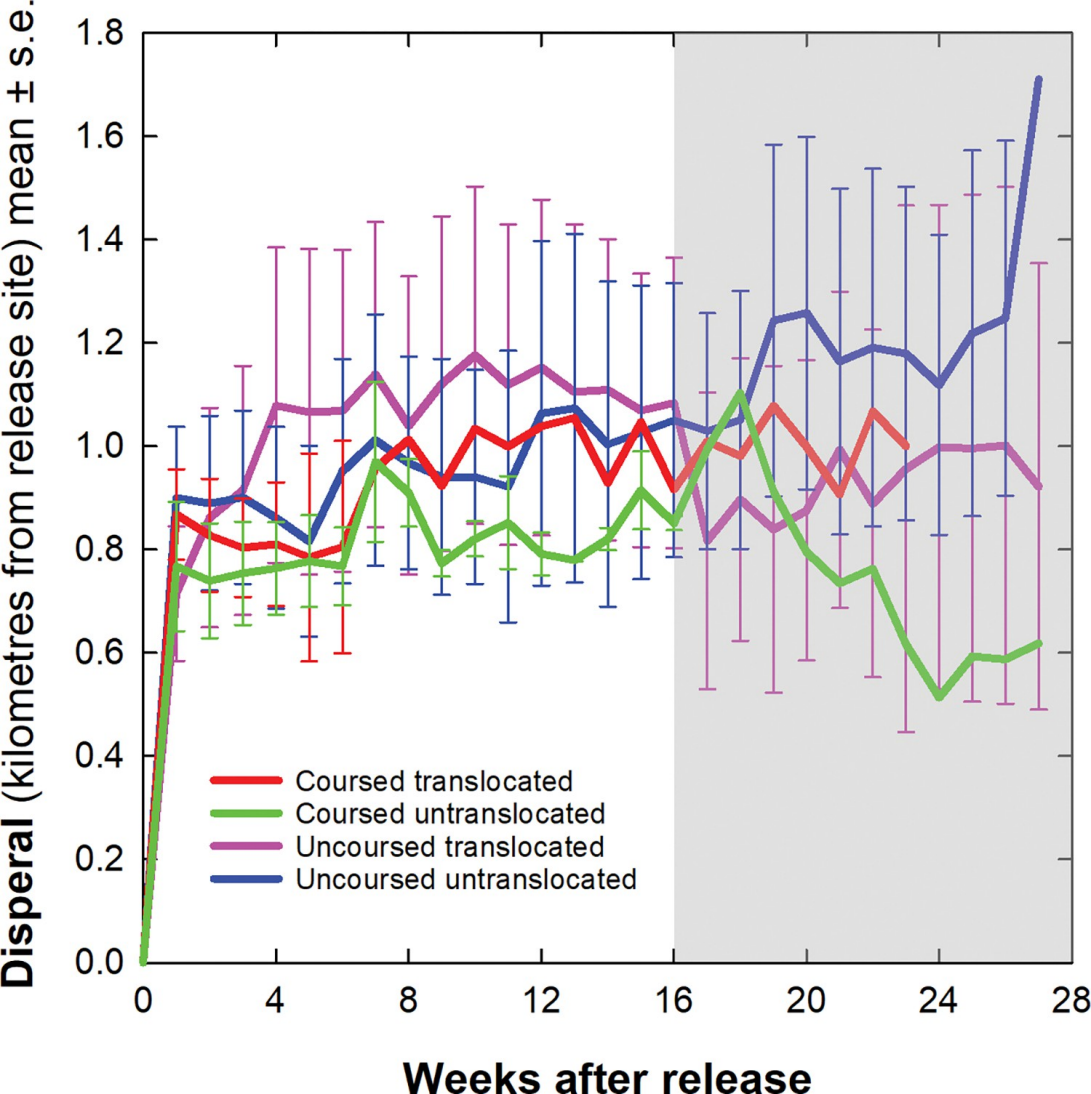
Hare dispersal distances over six months after release. Measured by the average distance from the release site each week in kilometres ± 1 standard error comparing all four cohorts of hares. The shaded area from Weeks 16–27 indicates caution should be used in interpreting results due to the small sample size of individual hares contributing spatial data.

Hares dispersed away from their respective release sites by around 800 (600–900) metres during the first week before drifting another 200m or so further over the next four months out to on average 1 (0.8–1.2) km (Week 1–16: *F*_df = 1,379_ = 59.179, *p*<0.001; Fig 6). Dispersal did not differ between the sexes (*F*_df = 1,220_ = 0.813, *p* = 0.368), coursed and uncoursed hares (*F*_df = 1,220_ = 0.784, *p* = 0.376), translocated and untranslocated hares (*F*_df = 1,220_ = 0.173, *p* = 0.678) or their interaction (*F*_df = 1,220_ = 0.004, *p* = 0.947; Fig 6).

## Discussion

This is the first study to attempt to assess the survival, movements and behaviour of coursed hares after their release back into the wild. Fewer coursed than uncoursed hares remained within the vicinity (within a few kilometres) of their release site six months later, not because more died (observed mortality did not differ between coursed and uncoursed cohorts), but a combination of translocated coursed hares removing their collars and radio silence from several untranslocated coursed hares. The coursed translocated cohort was the only group with no hares remaining on site five and a half months after release. Outcomes did not differ between translocated and untranslocated cohorts.

Five out of forty hares managed to slip their collar over their head with it remaining bolted closed indicating too loose a fit. There was no difference in the numbers that managed this between the experimental cohorts. There is always a trade-off between overtightening a collar to ensure fit and undertightening a collar to avoid causing the animal distress. In all cases, collars were fitted to leave space for two fingers to be comfortably slipped between the collar strap and the hare’s throat. In virtually all cases, collars were tightened to the same notch. Any loss of body condition throughout the early spring breeding season could conceivably have loosened collars.

Five out of forty, though 50% of the translocated coursed hares, removed their collars by fraying the straps until they wore through and failed usually within a month of release. This indicated an engineering failure where strap material was vulnerable to being compromised by hares scratching and gnawing. The supplier of the collars (LOTEK Ltd.) were informed and responded that no such problems had been reported by other researchers who had tagged similar hare species (for example, the European hare *L*. *europaeus*) with comparable collar models. All collars were from the same made-to-order batch and there is no reason to believe their quality should have varied between those deployed on different cohorts of hares. Why strap failures were not distributed randomly across the cohorts and clustered in the translocated coursed cohort is unknown. It was possibly not an effect of having been coursed as none of the collar straps failed on untranslocated coursed hares; and it was possibly not an effect of translocation as none of the collar straps failed on translocated uncoursed hares either. Whilst it could be speculated this behaviour may be the result of the combined effects of coursing and translocation (greater stress leading to lower tolerance for collar wearing) there was no evidence to support such a supposition and the role of random chance and statistical stochasticity cannot be ruled out when quantifying outcomes for cohorts of just ten individuals.

Eighteen out of forty hares were either never located after release, or, relocated but with subsequent loss of radio signal. The fate of undetected hares remains unknown; radio transmitter or antenna wiring may have failed, animals may have been predated with collars carried into underground burrows from where they would be undetected, or, animals may have dispersed rapidly in between radiotracking bouts beyond the search radius. For those animals successfully tracked, the average dispersal distance from the release site was only about 1km after several months. Irish hares are known to be relatively sedentary from previous tracking studies [12–14] with the current study providing no direct evidence for long distance dispersal i.e. no animals were located more than a few kilometres from the release sites despite wider searches.

Observed mortality did not differ between coursed and/or translocated hares. Three hares died during the study. Two (one translocated coursed and one untranslocated uncoursed) were killed in collisions with vehicles on their first evening after release. Both these cohorts were released at around 15:00 and 17:00 respectively (sunset was at 17:33) compared to 10:00 and 11:00 (sunrise was at 08:03) for translocated uncoursed and untranslocated coursed cohorts (in which no hares died). Most hares were released on the same day to standardise release dates and the difference in the timing of releases was due to the logistical constraints of travelling between sites and the time spent netting control animals. Licence conditions required that hares were released during daylight hours, which was the case, but the two cohorts with road kills on the first night had less daylight in which hares could settle before the onset of darkness having been released 30 minutes and 1.5 hours before sunset. This supports the current coursing licence condition that all hares should be released back into the wild during daylight hours (the recommendation here is as early as possible), to provide adequate time for them to settle in cover. All hares moved about twice as far on their first day than after they settled, increasing the risk of encountering dangers (so called, extrinsic sources of mortality), in this case road traffic. It was notable in both cases that the roads were busy straight stretches of National or Regional road with fast moving traffic such that at one site several other roadkill mammals were found suggesting it was a particularly dangerous crossing point for wildlife. Only one animal, a translocated coursed hare was confirmed as having been predated or scavenged by a fox due to evidence of diagnostic tooth marks and the remains of blood and flesh. Predation is to be expected and the death of a single hare cannot be attributed to this animal having been coursed and translocated more than five months earlier.

Three of the eight hares relocated alive at the end of the study, all uncoursed, were recaptured by the Irish Coursing Club during their normal netting operations for the next coursing season eight to nine and a half months after their initial release demonstrating survival. It is known from an earlier genetic fingerprinting study that individual hares were caught and tissued sampled across consecutive coursing seasons indicating interannual survival and release site philopatry [15].

In common with previous tracking studies [12–14], this study suggested that Irish hares have notably smaller home range sizes than other mountain hares in more typical mountain hare habitat (for example, up to 200 hectares in Finnish boreal forest [16]) but more comparable in their spatial ecology to European brown hares (*L*. *europaeus*) which have similar home range sizes in agricultural grasslands [17]). As the seasons progress from winter to summer the ratio of dark-to-light shifts in northern latitudes such that tracked Irish hares, like hares elsewhere, became more active during the day as the year progresses [18]; a trend that reversed after the solstice. There was no effect of coursing, translocation or their interaction on the apparent movements of hares during what would otherwise be their inactive period suggesting all hares lay up during the day in their forms with little or no appreciable variation in daylight behaviour across the cohorts.

All hares moved more during the first four days after release before settling, with translocated hares moving 1.5 times further, covering 3.8 times the area, of untranslocated controls. Most of this movement happened immediately after release whereupon animals settled into moving about 2km, and covering about 14 hectares, per day by Day 4 onwards. Most of this movement occurred during their active period one hour before sunset until one hour after sunrise. Irish hares are typically crepuscular with camera trapping data also showing them more active at sunrise than sunset [19]. Movements, home range size and dispersal of translocated and untranslocated hares did not differ after they had settled within the first few days after release, nor did they differ in mortality or relocation rates, suggesting the impacts of accidental translocation of hares in returning the same number of coursed animals, as were captured, to a release site, may be limited. Other studies that GPS-radio tracked herbivores (for example, big horn sheep *Ovis canadensis* or caribou *Rangifer tarandus caribou*) have also demonstrated little lasting effects of translocation with comparable survival to controls and little dispersal from release sites [20, 21].

## Recommendations

Any future investigation should consider the use of either cellular (e.g. GPS-GSM) or satellite (e.g. Argos) collars which relay locational data via mobile phone network or satellite connections without the need for radiotracking and the limitations of *in situ* data download. By way of example, satellite tags (KiwiSat303 collars from LOTEK Ltd.) were used recently to show that one Arctic hare (*L*. *arcticus*) in Greenland migrated 388km over 49 days [22] demonstrating similar technology may mitigate radio signal losses due to long distance dispersal. Any manufacturers of collars for deployment on Irish hares should be aware of the need for collar strap materials to be impervious to fraying to reduce strap failures.

## Conclusions

This work provides evidence to support current Government coursing licence conditions about where and when to release hares and provides the first assessment of coursed hare movements, home range size and dispersal after release back into the wild. Translocated hares moved further, and ranged over a larger area, for four days after release before settling into a pattern similar to other hares with no apparent consequence for their survival. Coursed and uncoursed hares did not differ in observed mortality, movements, home range sizes or dispersal distances after release back into the wild though fewer coursed than uncoursed hares were relocated six months after release, due to a combination of collar strap failures and radio silence. Any future study should consider cellular or satellite collars and stouter strap materials to minimise losses and maximise known outcomes.

## Supporting information

S1 Fig Accuracy of interpolating movements for missing GPS data. (PDF)

S2 Fig Photograph of hare collar strap failures. (PDF)

S3 Fig There was no relationship between outcomes and body weight. (PDF)

S1 Table Outcomes for forty individual hares six months after release. (PDF)
