# Supplementary material for: Survival, movements, home range size and dispersal of hares after coursing and/or translocation
Source: PLoS One. 2023 Jun 2;18(6):e0286771. doi: 10.1371/journal.pone.0286771 (PMC10237436; doi:10.1371/journal.pone.0286771)
Supplement: S1 Fig — (PDF) [file pone.0286771.s001.pdf]

Supporting Information

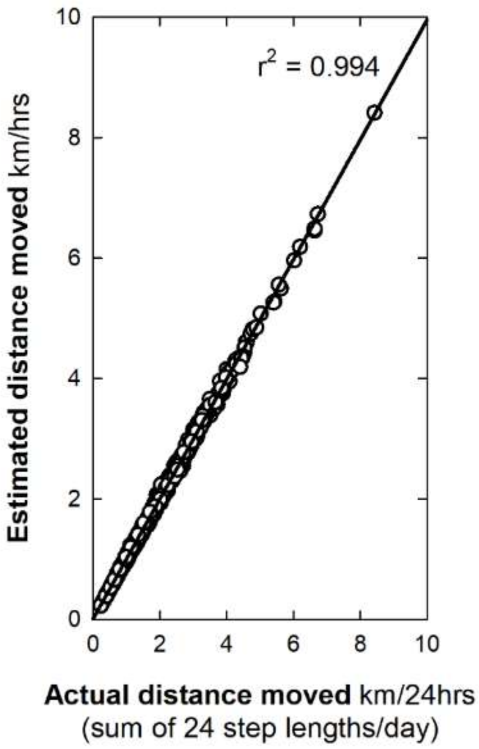

S1 Fig. Accuracy of interpolating movements for missing GPS data. Linear regression between the actual total summed distance moved by hares every 24 hours when 24 GPS fixes were successfully collected, and the distance estimated as the sum of the active and inactive periods' hourly averages multiplied by their duration each day.
