# Supplementary material for: Survival, movements, home range size and dispersal of hares after coursing and/or translocation
Source: PLoS One. 2023 Jun 2;18(6):e0286771. doi: 10.1371/journal.pone.0286771 (PMC10237436; doi:10.1371/journal.pone.0286771)
Supplement: S2 Fig — (PDF) [file pone.0286771.s002.pdf]

Supporting Information

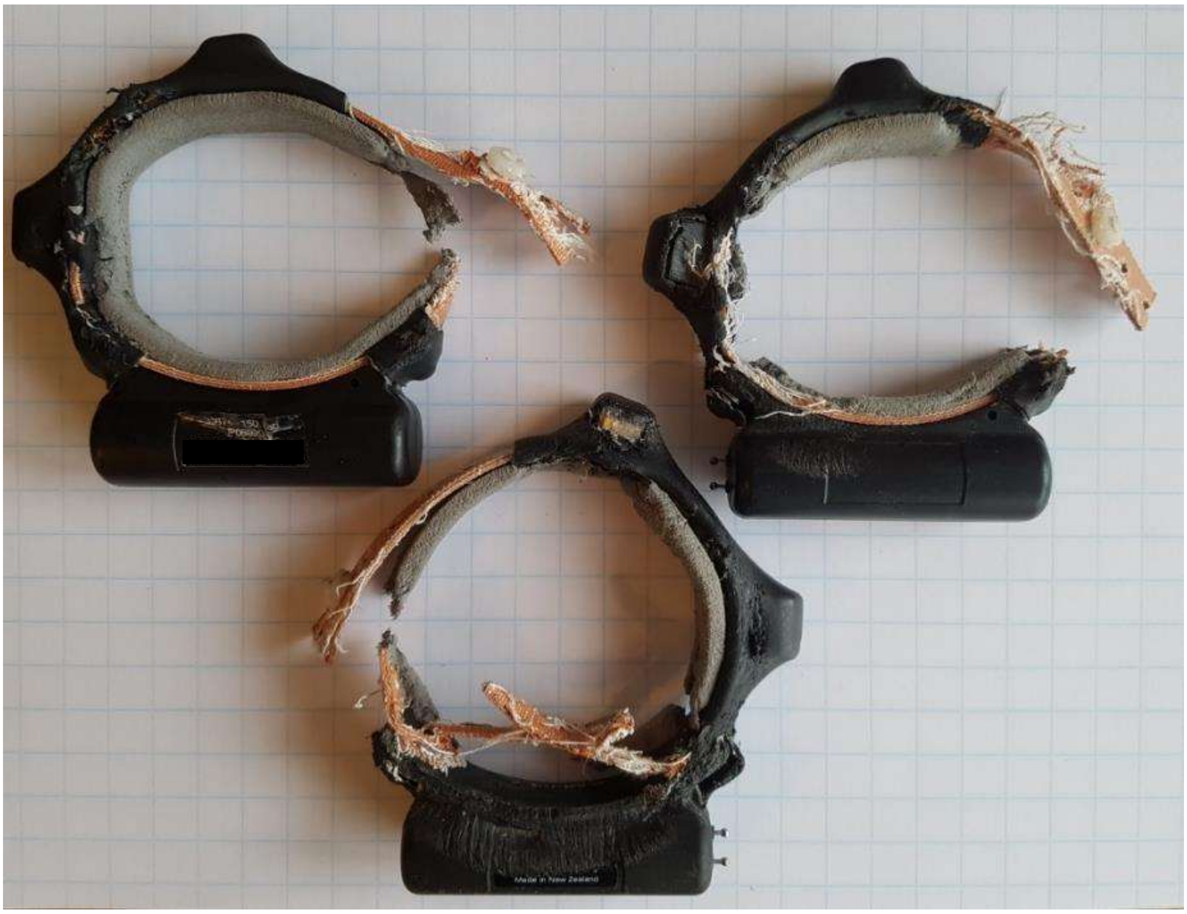

S2 Fig. Photograph of hare collar strap failures. Examples of three Litetrack RF60 collars (LOTEK Ltd.) where coursed translocated hares slashed through the strap with their hind nails with evidence of gnawing the payload barrel. There was no evidence of predation or other extrinsic sources of mortality.
