# Supplementary material for: Survival, movements, home range size and dispersal of hares after coursing and/or translocation
Source: PLoS One. 2023 Jun 2;18(6):e0286771. doi: 10.1371/journal.pone.0286771 (PMC10237436; doi:10.1371/journal.pone.0286771)
Supplement: S3 Fig — (PDF) [file pone.0286771.s003.pdf]

Supporting Information

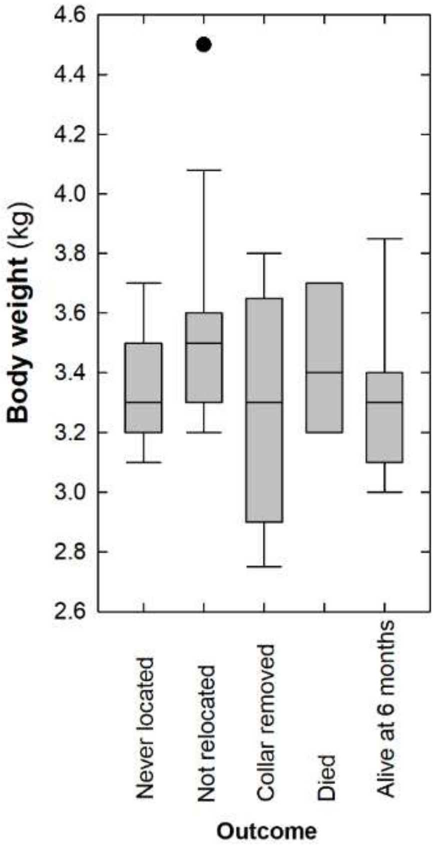

S4 Fig. There was no relationship between outcomes and body weight. Boxplot of hare body weights at release and outcomes six months later.
