# Supplementary material for: Survival, movements, home range size and dispersal of hares after coursing and/or translocation
Source: PLoS One. 2023 Jun 2;18(6):e0286771. doi: 10.1371/journal.pone.0286771 (PMC10237436; doi:10.1371/journal.pone.0286771)
Supplement: S1 Table — (PDF) [file pone.0286771.s004.pdf]

Supporting Information

S3 Table. Outcomes for forty individual hares six months after release. Forty individual hares showing the experimental cohort to which each belonged, their sex, weight and duration of tracking with the final outcome 6 months after release. Averages are given as means ± 1 standard deviation.

Cohort	Hare_ID	Sex	Weight (kg)	Release date (first data)	End date (last data)	Days tracked	Outcome at 6 months
Coursed translocated	33	M	3.7	08/02/2022	08/02/2022	0	Collar retrieved within <24hrs
	23	M	3.4	08/02/2022	09/02/2022	1	Dead. Road traffic collision.
	32	M	3.6	08/02/2022	11/02/2022	3	Collar retrieved
	25	M	3.3	08/02/2022	19/02/2022	11	Collar retrieved
	24	F	3.8	08/02/2022	24/02/2022	16	Not relocated
	27	M	3.4	08/02/2022	24/02/2022	16	Not relocated
	30	M	3.4	08/02/2022	04/03/2022	24	Collar retrieved
	26	F	3.8	08/02/2022	12/03/2022	32	Collar retrieved
	31	M	3.0	08/02/2022	24/03/2022	44	Collar retrieved
	28	F	3.7	08/02/2022	24/07/2022	166	Dead. Likely fox predation.
		**7M:3F**	**3.5 ± 0.3**			**31 ± 49**	
Coursed untranslocated	1	F	3.5	08/02/2022	08/02/2022	0	Never relocated after release
	4	M	3.1	08/02/2022	08/02/2022	0	Never relocated after release
	8	F	3.3	08/02/2022	08/02/2022	0	Never relocated after release
	9	M	3.1	08/02/2022	15/02/2022	7	Collar retrieved
	6	F	3.6	08/02/2022	26/02/2022	18	Not relocated
	10	M	3.2	08/02/2022	26/03/2022	46	Not relocated
	11	M	3.5	08/02/2022	26/03/2022	46	Not relocated
	7	M	3.3	08/02/2022	22/04/2022	73	Not relocated
	3	F	3.4	08/02/2022	03/06/2022	115	Not relocated
	5	M	3.4	08/02/2022	18/08/2022	191	Relocated alive at 6.3 months (study end)
		**6M:4F**	**3.4 ± 0.2**			**50 ± 62**	
Uncoursed untranslocated	34	M	3.4	08/02/2022	08/02/2022	0	Never relocated after release
	38	F	3.7	08/02/2022	08/02/2022	0	Never relocated after release
	39	F	3.2	08/02/2022	09/02/2022	1	Dead. Road traffic collision.
	36	F	3.6	08/02/2022	19/02/2022	11	Not relocated
	29	M	3.5	12/02/2022	01/03/2022	17	Collar retrieved
	40	M	2.8	12/02/2022	20/06/2022	128	Collar retrieved (inside round bale)
	41	M	3.2	12/02/2022	16/08/2022	185	Relocated alive at 6.1 months (study end)
	22	M	3.2	12/02/2022	16/08/2022	185	Relocated alive at 6.1 months (study end)
	35	M	3.5	08/02/2022	16/08/2022	189	Relocated alive at 6.2 months (study end)
	37	F	3.3	08/02/2022	08/10/2022	242	Recaught alive 8.0 months later (study end)
		**6M:4F**	**3.3 ± 0.5**			**96 ± 99**	
Uncoursed translocated	17	M	3.2	08/02/2022	08/02/2022	0	Never relocated after release
	20	M	3.3	12/02/2022	12/02/2022	0	Never relocated after release
	12	F	3.2	08/02/2022	25/02/2022	17	Not relocated
	16	F	2.6	08/02/2022	07/03/2022	27	Collar retrieved
	21	F	4.5	12/02/2022	03/06/2022	111	Not relocated
	18	M	3.4	08/02/2022	03/06/2022	115	Not relocated
	13	M	3.1	08/02/2022	18/08/2022	191	Relocated alive at 6.3 months (study end)
	14	M	3.9	08/02/2022	18/08/2022	191	Relocated alive at 6.3 months (study end)
	19	F	3.3	08/02/2022	10/10/2022	244	Recaught alive 8.0 months later (study end)
	15	M	3.0	08/02/2022	24/11/2022	289	Recaught alive 9.5 months later (study end)
		**6M:4F**	**3.3 ± 0.5**			**119 ± 107**	
